# Oral health-related quality of life in Tunisian patients following radiotherapy for head and neck tumors: a cross-sectional study

**DOI:** 10.11604/pamj.2025.52.26.47417

**Published:** 2025-09-18

**Authors:** Ghada Bouslama, Farah Raouani, Hanen Boukhris, Hajer Zidani, Lamia Oualha, Souha Ben Youssef

**Affiliations:** 1 Dentistry Department, Research Laboratory Functional and Aesthetic Rehabilitation of Maxillary LR12SP10, Farhat Hached Hospital, University of Sousse, Sousse, Tunisia

**Keywords:** Quality of life, radiotherapy, head and neck neoplasms, oral health, Tunisia

## Abstract

**Introduction:**

cervicofacial radiotherapy is a cornerstone in the management of head and neck tumors, utilizing ionizing radiation. Despite its efficacy, it can lead to significant side effects that impact patients´ Oral Health-related Quality of Life (OHRQoL) during and after treatment. This study aims to evaluate OHRQoL in Tunisian patients following cervicofacial radiotherapy and identify key factors influencing post-treatment quality of life.

**Methods:**

a multicenter, cross-sectional study was conducted across multiple hospitals in Tunisia. Twenty-five patients who underwent cervicofacial radiotherapy were included. Data collection involved a standardized questionnaire covering epidemiological data, radiotherapy details, oral health status, post-radiotherapy compliance, and OHRQoL assessment using the General Oral Health Assessment Index (GOHAI).

**Results:**

the findings revealed that 76% of patients underwent Intensity-Modulated Radiotherapy (IMRT), 54% used fluoride gel trays, and only 52% of patients had consulted a dentist in the past two years outside of routine follow-up appointments. Poor quality of life reported in 72% of patients was statistically correlated with age, general health status, dosimetry, chemotherapy, radiotherapy technique, and frequency of dental consultations.

**Conclusion:**

a significant proportion of patients experienced poor OHRQoL post-radiotherapy, influenced by factors such as age, radiation dose, radiotherapy technique, and dental visit frequency. IMRT was associated with better OHRQoL compared to conventional techniques. Limited compliance with oral health protocols underscores the need for enhanced pre- and post-radiotherapy dental care strategies, improved patient education, and increased accessibility to advanced radiotherapy techniques in Tunisia. Larger studies with extended follow-up are necessary to validate these findings and enhance post-radiotherapy oral healthcare policies.

## Introduction

Head and neck cancers represent over 4.5% of global cancer cases, posing a significant public health challenge [1]. The successful treatment of these malignancies requires a multidisciplinary strategy, where radiation therapy serves as a central component, often combined with surgical intervention and chemotherapy [2]. The development of intensity-modulated radiotherapy (IMRT) has significantly improved treatment outcomes by delivering targeted high-dose radiation to tumor locations while minimizing damage to surrounding healthy tissues [3]. Despite these advancements, radiotherapy can have adverse effects that significantly impact patients' Oral Health-related Quality of Life (OHRQoL), which includes functional limitations (such as difficulty chewing or speaking), physical discomfort, psychological distress, and social impairment [4]. Identifying factors that affect OHRQoL in patients who have undergone radiation therapy for head and neck tumors is crucial for optimizing patient care and long-term outcomes.

While numerous studies have explored this topic across various populations [5-7], there is a notable lack of data from Tunisia and the broader North African region. This gap limits the applicability of existing findings, as healthcare systems and patient support may differ significantly from those in high-income countries. In Tunisia, disparities in the availability of oral supportive care and patient follow-up could impact the severity and management of treatment-related side effects. Additionally, recent epidemiological data show that head and neck tumors now make up approximately 12% of all cancer cases in the country [8], highlighting the need to address their broader impact on patient well-being.

Given this context, the present study aims to evaluate the OHRQoL of Tunisian patients who have undergone radiotherapy for head and neck tumors and identify factors that may negatively affect their quality of life. By addressing a significant regional knowledge gap, this research seeks to provide insights for improved post-radiotherapy management and patient-centered care strategies.

## Methods

**Study design and setting:** this study is a multicenter, cross-sectional observational study designed to capture a snapshot of OHRQoL in patients following cervicofacial radiotherapy. The study was conducted over four months, from December 2024 to March 2025. Participant recruitment was conducted across several hospitals in northern and central Tunisia (Tunis and Sousse) to ensure geographic and demographic diversity. The participating institutions were: i) the Department of Dental Medicine at Charles Nicolle Hospital in Tunis, a major public tertiary care center; ii) the Otolaryngology (ENT) Department at Salah Azaiez Hospital, a national referral center for oncology; iii) the Radiotherapy Department at Farhat Hached Hospital in Sousse, a regional cancer treatment facility; iv) the Dental Medicine Department at the same hospital. These hospitals were selected for their specialization in cancer treatment and accessibility to a wide spectrum of patients from both urban and rural areas.

The study was conducted in accordance with the Declaration of Helsinki. Ethical approval was obtained from the University Ethics Committee. Informed consent was obtained from all participants before data collection.

**Study population:** included Tunisian adults receiving treatment in public healthcare facilities who had completed cervicofacial radiotherapy for head and neck cancers. Participants were recruited using a convenience sampling method from outpatient consultations or scheduled follow-up visits, provided they met the inclusion criteria and agreed to participate during the study period. The sample size was estimated based on the number of eligible patients attending the selected hospitals during the four months. A formal calculation using the Cochran formula (with a 95% confidence level and an estimated response rate of 50%) was used to guide the minimum required sample size. All patients who met the inclusion criteria and provided informed consent were enrolled.

**Inclusion criteria:** i) patients with a confirmed history of cervicofacial radiotherapy with a minimum radiation dose of 40 Gy; ii) radiation fields involving oral cavity structures (maxilla or mandible); iii) at least six months´ post-radiotherapy to allow assessment of late effects; iv) ability to understand and complete the questionnaire.

**Exclusion criteria:** i) patients with incomplete medical records; ii) cognitive impairments affecting memory, comprehension, or language that interfered with accurate questionnaire completion; iii) patients currently undergoing active cancer treatment.

**Data collection:** data was collected using a standardized questionnaire. The data collection methods included patient interviews and clinical oral examinations conducted by the same postgraduate dental practitioner. The questionnaire was administered during in-person post-radiotherapy follow-up visits, structured into three main sections: i) Epidemiological and radiotherapy data: tumor location, irradiated area, radiation fractionation, total duration, dosimetry, and specification of whether radiotherapy was exclusive or combined with surgical resection and/or chemotherapy. ii) Oral health status and post-radiotherapy compliance: clinical oral hygiene examination: frequency of dental consultations, tooth brushing, and general dental status, periodontal health: plaque index assessment using the Silness-Löe plaque index and gingival inflammation evaluation using the clinical probing technique, post-radiotherapy compliance was assessed based on three criteria: frequency of dental visits, possession and use of fluoride trays, and adherence to oral hygiene practices. iii) Oral health-related quality of life assessment: through the GOHAI questionnaire, a validated tool assessing OHRQoL, including 12 items and covering three domains: functional, psychosocial, and pain/discomfort [9]. The scoring system was as follows: GOHAI < 50: Poor OHRQoL, 51 ≤ GOHAI < 56: Moderate OHRQoL, GOHAI > 57: Good OHRQoL.

**Statistical analysis:** descriptive statistics were used to summarize patient characteristics, treatment variables, and oral health-related quality of life (OHRQoL) scores. Categorical variables such as age group, radiation dose, radiotherapy technique, and time since the last dental visit were compared across OHRQoL categories (poor, moderate, good) using Fisher's exact test. A p-value of <0.05 was considered statistically significant. IBM SPSS Statistics version 23 was used for the analysis. No missing data were encountered for any of the variables included in this study.

## Results

**Epidemiological data:** a total of 25 participants, consisting of 18 males and 7 females, responded to the questionnaire. The mean age of participants was 55.6 years, ranging from 18 to 76 years old. In terms of general health, 13 participants (52%) reported being in good condition, while 4 (16%) had hypertension, 3 (12%) had diabetes, and 3 (12%) had hypothyroidism. Additionally, 1 participant had Crohn's disease and 1 had depression. The distribution of cervicofacial tumors among participants varied significantly. The most common diagnosis was nasopharyngeal carcinoma (13 cases, 52%), followed by squamous cell carcinoma of the larynx (4 cases, 16%), and mucoepidermoid carcinoma of the parotid gland (2 cases, 8%). Other tumor types, each observed in 1 participant (4%), included basal cell carcinoma of the orbit, tracheal carcinoma, squamous cell carcinoma of the external auditory canal, squamous cell carcinoma of the glottic and subglottic regions, squamous cell carcinoma of the lower gingiva, and pituitary adenoma. Regarding radiotherapy techniques, 19 patients (76%) received intensity-modulated radiotherapy (IMRT), 5 (20%) underwent conventional external beam radiotherapy, and 1 (4%) received 3D conformal radiotherapy (3D-CRT). More than half of the patients (13/25, 52%) received an irradiation dose between 60 and 70 Gy. Chemotherapy was administered to 15 participants (60%), and 16 patients (64%) underwent surgical excision following radiotherapy. Nine patients (36%) did not undergo tumor resection surgery before radiotherapy.

**Oral health status and post-radiotherapy compliance:** dental consultations varied among participants. Eleven participants (44%) had not visited a dentist in more than two years, and 1 participant (4%) had never visited a dentist. Among the remaining participants, 14 (56%) visited a dentist less than once per year, 9 (36%) had annual visits, and 2 (8%) consulted twice per year. Knowledge and use of fluoride trays were also assessed. More than half (54%) of the participants did not possess a fluoride tray, while only 6 (24%) were aware of its benefits, citing reasons such as 'protection of teeth', 'prevention of dental caries', and 'mitigation of radiation side effects.'

Clinical assessments were performed by a calibrated dental examiner using the Silness-Löe plaque index. Results showed that 7 participants (28%) had abundant plaque in interdental spaces, and 3 (12%) had no visible plaque. Among edentulous patients, the proportion with detectable plaque was comparable to that of those with moderate plaque levels. Gingival inflammation was evaluated using the clinical probing technique. Moderate inflammation with bleeding on probing and severe inflammation with spontaneous bleeding were noted in 7 cases (28%). Only 2 participants (8%) had healthy gingiva without signs of bleeding. Xerostomia was reported by 24 patients (96%) following treatment. Only 1 participant reported using a mouth spray. In terms of taste disorders, 10 participants (40%) occasionally experienced dysgeusia, 9 (36%) reported hypogeusia, and 6 (24%) experienced ageusia. Associations between oral health indicators and treatment-related factors were explored. Participants with longer intervals between dental visits and those not using fluoride trays tended to have higher plaque scores and more severe gingival inflammation.

**Comparative analysis based on oral health-related quality of life:** according to the GOHAI score, 18 participants (72%) experienced poor oral health-related quality of life (OHRQoL) following cervicofacial radiotherapy. Moderate OHRQoL was observed in 5 participants (20%), while only 2 participants (8%) reported good OHRQoL. The relationship between OHRQoL and clinical categorical variables was evaluated using Fisher's exact test, which was deemed more appropriate than the Chi-square test due to small cell counts (< 5). Poor OHRQoL was significantly more frequent in older age groups, particularly in patients over 40 years of age (p = 0.0001). In contrast, good OHRQoL was significantly more common in younger patients, with only one patient reporting good OHRQoL in the 0-20 age group (p = 0.044). No significant association was observed between age and moderate OHRQoL (p = 0.353).

A statistically significant association was found between both radiotherapy type and radiation dose with poor quality of life. Patients treated with conventional radiotherapy experienced worse OHRQoL outcomes compared to those receiving IMRT (p = 0.001). Similarly, a significant relationship between higher radiation doses and poor QoL (p = 0.001) was observed, with no significant associations found for moderate (p = 0.514) or good (p = 0.751) OHRQoL categories. The timing of the last dental visit was another influencing factor. Patients who had never consulted a dentist or had not done so in over two years were more likely to report poor OHRQoL. The distribution of OHRQoL scores based on the timing of the last dental visit was statistically significant (p = 0.045).

The statistical analysis indicated that variables associated with poor OHRQoL included older age (particularly over 40 years), the use of conventional radiotherapy techniques, higher radiation doses, and infrequent dental consultations. Younger patients and those receiving IMRT tended to report better OHRQoL. Table 1 shows the significant associations between poor OHRQoL and patient age, radiation dose, and other variables.

**Table 1:** associations between poor OHRQoL and patient age, radiation dose, radiotherapy techniques, and dental visits

Variable	Category	Poor QoL n (%)	Moderate QoL n (%)	Good QoL n (%)	Total (n)	p-value
**Age (Years)**	0-20	0 (0%)	0 (0%)	1 (100%)	1	-
**-**	20-40	2 (100%)	0 (0%)	0 (0%)	2	0.0001**
**-**	40-60	7 (63.6%)	3 (27.3%)	1 (9.1%)	11	-
**-**	60-80	9 (81.8%)	2 (18.2%)	0 (0%)	11	-
**-**	>80	1 (100%)	0 (0%)	0 (0%)	1	-
**Radiotherapy type**	Conventional RT	5 (100%)	0 (0%)	0 (0%)	5	0.001**
**-**	Conformal RT	0 (0%)	1 (100%)	0 (0%)	1	-
**-**	IMRT	13 (68.4%)	4 (21.1%)	2 (10.5%)	19	-
**Radiation dose (Gy)**	<30	0 (0%)	0 (0%)	0 (0%)	0	-
**-**	30-40	1 (50%)	1 (50%)	0 (0%)	2	0.001**
**-**	40-60	6 (66.7%)	2 (22.2%)	1 (11.1%)	9	-
**-**	60-70	10 (76.9%)	2 (15.4%)	1 (7.7%)	13	-
**-**	>70	1 (100%)	0 (0%)	0 (0%)	1	-
**Duration of condition**	Never	2 (100%)	0 (0%)	0 (0%)	2	0.045*
**-**	More than 2 years	9 (75%)	3 (25%)	0 (0%)	12	-
**-**	1-2 years	2 (100%)	0 (0%)	0 (0%)	2	-
**-**	6 months - 1 year	2 (50%)	1 (25%)	1 (25%)	4	-
**-**	Less than 6 months	4 (57.14%)	2 (28.57%)	1 (14.28%)	7	-

* significant at 5% level; **: highly significant; ns: not significant

## Discussion

This study aimed to evaluate the oral health-related quality of life (OHRQoL) in patients who underwent radiotherapy for head and neck tumors in Tunisia and identify factors that may negatively impact their OHRQoL.

**Summary of key findings:** the results revealed that 18 participants experienced poor OHRQoL post-radiotherapy, with the most commonly reported issues being xerostomia and taste disorders. OHRQoL in head and neck cancer patients plays a crucial role in communication, nutrition, and respiration. Many patients reported psychosocial and functional difficulties, including masticatory impairment, which may lead to nutritional deficiencies, dysphagia, anorexia, and long-term depression [10,11]. Several variables were found to significantly influence OHRQoL in this cohort, including age, radiation dose, radiotherapy technique, and frequency of dental visits. Specifically, older age was associated with poorer OHRQoL, likely due to age-related decline in salivary gland function, reduced tissue regeneration capacity, and decreased adherence to oral hygiene protocols. Higher radiation doses may exacerbate damage to salivary glands and oral mucosa, while infrequent dental visits reduce opportunities for early detection and management of oral complications, worsening patients' oral outcomes. This aligns with data demonstrating IMRT´s advantages in preserving adjacent healthy tissues while effectively targeting complex tumor regions [12]. IMRT has been associated with improved salivary gland function and better dental outcomes, thereby enhancing OHRQoL [13,14]. However, access to IMRT remains limited in Tunisian public hospitals due to equipment shortages and resource constraints. This disparity may lead to unequal care outcomes across regions and private hospitals. Comparative studies from low and middle-income countries with similar healthcare infrastructures, such as Algeria or Egypt, also highlight significant challenges in IMRT implementation. In contrast, European countries with broader IMRT availability report more favorable post-treatment OHRQoL, suggesting that technological access and better funding for the public health sector may explain some discrepancies between regions [15,16].

Our study also revealed that poor oral hygiene practices, including high plaque and gingival indices, were prevalent among participants. Notably, many patients had not visited a dentist in over two years, and some had never received professional dental care. This lack of preventive care was associated with worsened OHRQoL. Furthermore, compliance with fluoride prophylaxis was low, an essential component in preventing radiation-induced dental caries. Savignat *et al*. similarly found that more than half of their patient population did not adhere to prophylactic protocols, with only 43% demonstrating full compliance [17]. In our context, low adherence may stem from a combination of factors, including inadequate awareness, discomfort from poorly fitting fluoride trays, lack of motivation, and limited use of fluoride gel due to the limited financial resources of public hospital patients.

**Comparison with previous studies:** our findings align with previous international studies that have emphasized the significant negative impact of radiotherapy on OHRQoL among head and neck cancer patients [18,19]. Similar to reports from Sri Lanka, Brazil, Norway, Pakistan, and India, our patients often experienced xerostomia, dysgeusia, and difficulties in chewing and maintaining oral hygiene [20-24]. Notably, studies from Brazil and India indicated that patients treated with intensity-modulated radiotherapy (IMRT) reported better OHRQoL outcomes, which is consistent with our observation of poorer outcomes in patients who received conventional radiotherapy [21,23]. Furthermore, like the Norwegian and Pakistani studies, our results underscore the link between infrequent dental visits and decreased OHRQoL [22,24]. However, our study uniquely highlights systemic barriers specific to the Tunisian context, such as limited access to IMRT and the absence of preventive dental care integration into oncology protocols.

**Study limitations:** this study has several limitations that should be acknowledged to enhance transparency and provide context for the findings. Firstly, the relatively small sample size limits the generalizability of the results to the broader Tunisian population. This limitation was primarily due to the short, four-month recruitment period and challenges in patient enrollment, including poor adherence to post-radiotherapy follow-up appointments and exclusions based on incomplete medical records. Secondly, the use of self-reported data introduces the risk of recall bias and subjective misreporting. Additionally, the absence of a control group further restricts the ability to draw causal inferences. Finally, the study was conducted exclusively in public healthcare institutions, which may not fully represent patients treated in private healthcare settings.

**Clinical implications and recommendations for future practice:** despite these limitations, our findings highlight critical gaps in the post-radiotherapy care of head and neck cancer patients in Tunisia. These include inadequate oral hygiene maintenance, limited access to advanced radiotherapy techniques in public health hospitals, and poor adherence to preventive dental care protocols. To address these issues, we propose the following: i) Expand IMRT access: pilot programs could be introduced not only in major public university hospitals but also in other healthcare facilities, prioritizing patients with nasopharyngeal or oropharyngeal tumors. These programs should be accompanied by staff training and cost-benefit assessments to ensure sustainability. ii) Integrate dental care into oncology services: establish mandatory pre-radiotherapy dental screenings and continuous dental follow-up protocols as part of national cancer treatment guidelines. iii) Enhance patient education and fluoride prophylaxis: develop patient-centered education initiatives to improve awareness and motivation. Distribute subsidized fluoride trays with a better design and comfort, especially in public hospitals. By addressing these areas, Tunisian healthcare services can work toward improving long-term outcomes and enhancing OHRQoL for head and neck cancer survivors.

## Conclusion

This study provided valuable insights into the oral health-related quality of life (OHRQoL) of patients in Tunisia who have undergone radiotherapy for head and neck cancers. The findings revealed that 18 participants (72%) experienced poor OHRQoL, with xerostomia and taste disorders being the most frequently reported symptoms. Key factors significantly associated with reduced OHRQoL included older age, higher radiation dose, use of conventional radiotherapy techniques, and infrequent dental consultations. By evaluating these associations, the study successfully met its original objective of identifying clinical and behavioral variables that negatively impact OHRQoL. These findings underscore the critical need for comprehensive, multidisciplinary care that includes routine pre-radiotherapy dental assessments and continuous dental follow-up. For policymakers, the results support expanding access to advanced radiotherapy techniques such as IMRT, particularly in public hospitals where such technologies remain limited. Future research should focus on longitudinal studies to monitor changes in OHRQoL over time, assess the impact of targeted interventions (such as improved fluoride tray distribution and education programs), and compare outcomes across public and private healthcare settings to identify disparities and best practices. These steps are essential for developing tailored, evidence-based strategies to enhance the quality of life for head and neck cancer survivors in Tunisia and similar resource-limited contexts.

### What is known about this topic

- Radiotherapy for head and neck tumors has a profound impact on OHRQoL, affecting both the physical and psychological well-being of patients;
- The development of intensity-modulated radiotherapy (IMRT) has significantly improved OHRQoL scores after cervicofacial radiotherapy.

### What this study adds

- This study presents the first comprehensive evaluation of oral health-related quality of life (OHRQoL) in Tunisian patients undergoing radiotherapy for head and neck tumors, addressing a significant gap in regional data;
- More than half of the participants reported poor OHRQoL, with xerostomia and taste disorders being the most frequent complaints;
- The study also underscores systemic challenges specific to Tunisia, such as limited access to intensity-modulated radiotherapy (IMRT) in public hospitals and the lack of integrated preventive dental care within oncology protocols.
